# Effect of the Camelid’s Seminal Plasma Ovulation-Inducing Factor/β-NGF: A Kisspeptin Target Hypothesis

**DOI:** 10.3389/fvets.2017.00099

**Published:** 2017-06-30

**Authors:** Khalid El Allali, Najlae El Bousmaki, Hassan Ainani, Valérie Simonneaux

**Affiliations:** ^1^Comparative Anatomy Unit/URAC49, Department of Biological and Pharmaceutical Veterinary Sciences, Hassan II Agronomy and Veterinary Medicine Institute, Rabat, Morocco; ^2^Department of Neurobiology of Rhythms, CNRS UPR 3212, Institute of Cellular and Integrative Neurosciences, University of Strasbourg, Strasbourg, France

**Keywords:** ovulation-inducing factor, β-NGF, camelids, camel, ovulation, seminal plasma, kisspeptin

## Abstract

Female mammals are classified into spontaneous and induced ovulators based on the mechanism eliciting ovulation. Ovulation in spontaneous species (e.g., human, sheep, cattle, horse, pigs, and most rodents) occurs at regular intervals and depends upon the circulating estradiol. However, in induced ovulators (e.g., rabbits, ferrets, cats, and camelids), ovulation is associated with coitus. In the later, various factors have been proposed to trigger ovulation, including auditory, visual, olfactory, and mechanic stimuli. However, other studies have identified a biochemical component in the semen of induced ovulators responsible for the induction of ovulation and named accordingly ovulation-inducing factor (OIF). In camelids, intramuscular or intrauterine administration of seminal plasma (SP) was shown to induce the preovulatory luteinizing hormone (LH) surge followed by ovulation and subsequent formation of corpus luteum. Recently, this OIF has been identified from SP as a neurotrophin, the β subunit of nerve growth factor (β-NGF). β-NGF is well known as promoting neuron survival and growth, but in this case, it appears to induce ovulation through an endocrine mode of action. Indeed, β-NGF may be absorbed through the endometrium to be conveyed, *via* the blood stream, to the central structures regulating the LH preovulatory surge. In this review, we provide a summary of the most relevant results obtained in the field, and we propose a working hypothesis for the central action of β-NGF based on our recent demonstration of the presence of neurons expressing kisspeptin, a potent stimulator of GnRH/LH, in the camel hypothalamus.

## Introduction

Ovulation is a critical event in the reproductive function in all female mammals. Gonadotropin-releasing hormone (GnRH) is the key regulatory neuroendocrine pathway implicated in the regulation of ovulation. Based on the biological process that triggers the release of GnRH, two categories of species are classified as follows: spontaneous and induced ovulators. In animals considered as spontaneous ovulators (e.g., women, cattle, horses, sheep, goat, pigs, monkeys, and most rodents), ovulation occurs at regular intervals. The ovarian follicular dynamic leads to the emergence of one or more dominant follicles (depending of species been monoparous or multiparous), which increases the systemic concentration of estradiol. High estradiol concentration switches its negative to a positive feedback, which is permissive for the activation of GnRH release into the portal blood. Recent studies have demonstrated that hypothalamic neurons expressing kisspeptin (Kp) are critical to integrate the negative-to-positive switching effect of estradiol and to activate GnRH release (1). GnRH in turn triggers a strong and transitory release of luteinizing hormone (LH) (LH surge) from the gonadotrophs of the pituitary gland (2). This marked increase in circulating LH activates a whole cascade of inflammatory and proteolytic responses leading to the rupture of the dominant follicular boundary wall and the extrusion of the oocyte with its *cumulus oophorus* (3–5). In induced (also called reflex) ovulators (e.g., rabbit, Bactrian and dromedary camel, llama, alpaca, cat, ferret, etc.), the signals generated by copulatory stimulation during mating appear sufficient to induce ovulation while the positive feedback action of estradiol is reduced or absent (6, 7). It has been suggested that genital-somatosensory signals generated by penile intromission during copulation activate neural circuitries, mainly noradrenergic neurons, in the midbrain and brainstem to promote GnRH release (6). Several other stimuli including emotional, olfactory, auditory, visual, and tactile signals have also been assumed to facilitate ovulation (8, 9). The purification of a biochemical component from camelid’s semen, which was able to trigger ovulation, led to the concept of an “ovulation-inducing factor (OIF)” in the seminal plasma (SP) of induced ovulators (10, 11). The existence of this OIF was then confirmed in several other studies (12–16) and recently identified by Ratto et al. (17) as a neurotrophin: β subunit of nerve growth factor (β-NGF).

This review presents the history and discovery of OIF/β-NGF, highlights recent findings regarding its biochemical identification and sites of action and proposes hypothesis for the mechanisms through which the seminal β-NGF acts on the central nervous systems of female induced ovulators, mainly camelids.

## OIF in the SP of Camelids Breaks Old Dogma

The understanding of the mechanism by which ovulation is initiated in induced ovulators was the purpose of different studies since the 1960s. The first evidence of the existence of an induced process of ovulation in camelids was reported in alpacas where authors documented that mounting accompanied by intromission was necessary to provide adequate stimulation of LH release and the subsequent ovulation (8, 18, 19). Later on, Shalash and Nawito (20) observed that ovulation also required mating in dromedary and Bactrian camels and suggested that coitus, mechanical or electrical stimuli of the cervix would be essential for ovulation. From these early studies, it was believed that physical stimulation of the genitalia during copulation is the primary trigger for inducing ovulation in camelids species. This dogma lasted until 1985 when two remarkable studies published in the same *Journal of Reproduction and Fertility* (10, 11) reported the ovulation-inducing effect of semen in Bactrian camel. Both studies showed that the single intramuscular or intravaginal administration of male SP induced a surge in LH followed by ovulation. In their study, Chen et al. (10) compared the effect of intravaginal administration of (1) fresh and frozen semen, (2) SP, (3) washed spermatozoa, and (4) intramuscular injection of SP of bull, on ovulation rate in female Bactrian susceptible to ovulate (follicles diameter ≥1.2 cm). Their results showed that ovulation occurred in 28/32 females inseminated with whole semen, independently of whether the semen contained or not frozen spermatozoa. Contrastingly, none of the female inseminated with washed spermatozoa ovulated. Furthermore, ovulation was observed in 6/8 females inseminated with Bactrian SP and in 2/7 female injected with bull SP. In all case, the timing of ovulation was similar to that observed following natural mating (36–48 h later). In the other study, Xu et al. (11) found similar results and further reported that 4 h after intravaginal deposition of semen, an LH surge occurred followed by ovulation 36–48 h later, thus indicating that the semen-induced ovulation depends upon an LH surge.

Further studies, especially in Chile and Canada, confirmed the robust ovulatory effect of SP in llamas and alpacas. A single intramuscular dose of SP (<1/4 of an ejaculate) of llamas or alpacas induced an increase in plasma LH concentrations and ovulation in more than 90% of females (13). In a recent work, Berland et al. (21) not only confirmed that intrauterine infusion of 5 ml SP induces ovulation in llamas but also that ovulation depends more on this chemical signals than on physical stimulation or penile intromission as ovulation rate was of 0% when mating was performed by urethrostomized male, as compared to 85.7% when mating was done by intact male.

From these studies, authors suggested the presence of a potent factor in the SP of induced ovulators responsible for the induction of ovulation and named it accordingly OIF or “GnRH-like factor.” This concept was gradually accepted and many scientists then suggested that induced ovulation is a multifactorial event occurring as a consequence of several signals: mechanical stimuli of coitus, male effect, and most importantly OIF in the SP (22–25).

## Comparative Approach of the OIF Versus GnRH Effects

**Figure 1:**
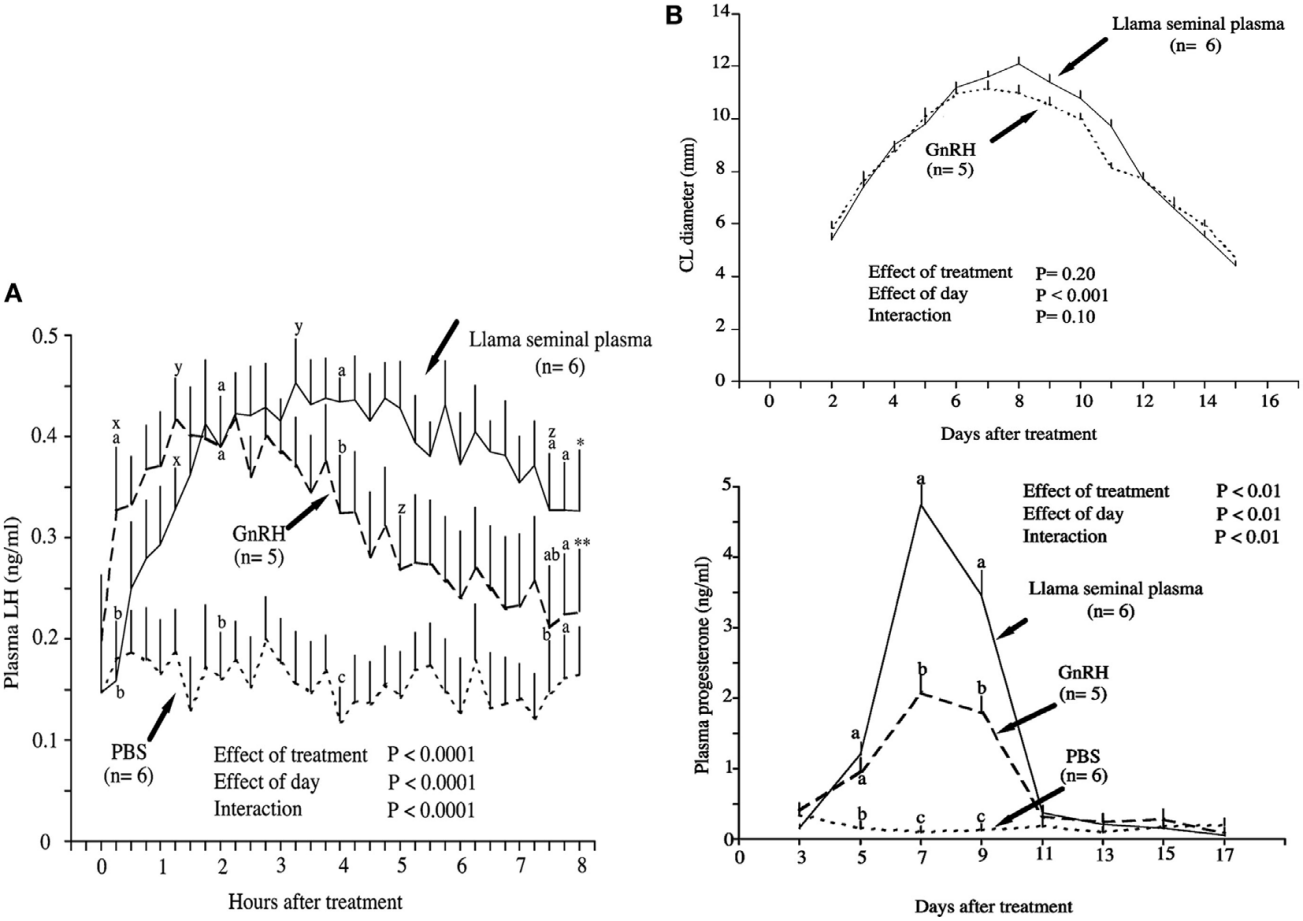
Effect of intramuscular treatment of female llamas with llama seminal plasma, gonadotropin-releasing hormone (GnRH), or phosphate-buffered saline (PBS). The effect was studied on **(A)** plasma luteinizing hormone concentrations and **(B)** corpus luteum diameter and plasma progesterone concentrations. Data are represented as mean ± SEM. ^a,b,c^On a given day, values with no common superscript are different among groups (*P* < 0.05). ^x^Within group, the first increase from pretreatment (time 0) concentration (*P* < 0.05). ^y^Within group, the maximum concentration (*P* < 0.05). ^z^Within group, the first decrease from maximum concentration (*P* < 0.05). *Within group, the last value is higher than the pretreatment value (*P* < 0.05). **Within group, the last value is not different from the pretreatment value (*P* = 0.9) [modified from Ref. (13)].

So far, the described effect of OIF appears similar to that of GnRH used for the control of reproduction in camelids (26, 27) leading to the question of similar mechanisms of action of both molecules. A comparative study performed by Adams et al. (13) in female llama showed that intramuscular injection of SP induced a higher ovulation rate (93%) than GnRH (83%). Interestingly, the increase in plasma LH concentration observed in SP treated female was faster but more prolonged as compared to the ones treated by GnRH: a significant increase in plasma LH concentration was seen 15 min after treatment with SP and 75 min after injection of GnRH; the maximum was reached after 2 h with SP as compared to 1 h with GnRH; and finally, the decrease was delayed by 2.5 h in the SP treated group (Figure 1A). Furthermore, the corpus luteum (CL) showed a greater diameter, regressed later, and produced more than two times progesterone in SP treated group as compared to the GnRH injected group (Figure 1B). Interestingly, the pattern of SP-induced LH surge (13) is very similar to that described in response to natural mating (28, 29). A careful reading of figures of another study comparing the effect of purified OIF to a GnRH analog (Buserelin) in dromedary females (30) showed that progesterone secretion subsequent to ovulation is prolonged in OIF-treated group as compared to GnRH analog group. This seems to indicate that in female camel also, the luteotrophic activity of OIF is more important than that of GnRH.

Different studies (31–33) demonstrated that as for GnRH, the OIF shows a dose–response effect on circulating concentration of LH and the incidence of ovulation in llamas and alpaca. However, unlike GnRH, purified OIF showed a dose–response relationship for the CL diameter and plasma progesterone concentrations (31, 33). This indicates a possible luteotrophic effect of OIF, which is independent of the mechanisms involved by GnRH.

The important luteogenesis effect of OIF/SP can be explained by its local effect at the level of the CL. Indeed, it was reported that its administration induces an important angiogenesis with a high blood flow, promoting the formation of the CL (34–36). Altogether, these results indicate that OIF and GnRH are two different molecules because OIF effect is more important than that of GnRH and they affect pituitary LH release differently.

## Isolation and Purification of the OIF

**Figure 2:**
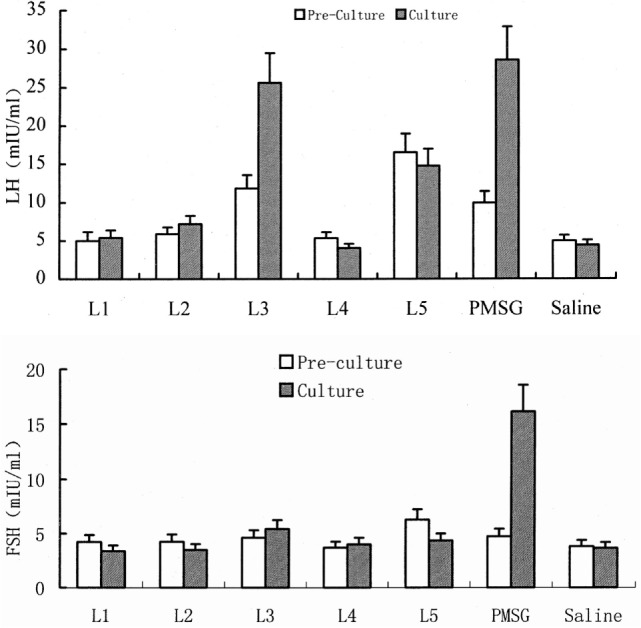
Effect of different protein fractions separated from camel seminal plasma on luteinizing hormone (LH) and FSH concentrations in pre-culture and culture of rat pituitary tissue. Here, the example for L3 shows that this fraction increases significantly LH concentration as well as pregnant mare’s serum gonadotropin (PMSG) but has no effect on FSH concentrations [modified from Ref. (12), ©2001 Blackwell Wissenschafts-Verlag, Berlin].

Important effort has been conducted to characterize the biochemical nature of the semen OIF. A preliminary study carried out in Bactrian camel reported that OIF bioactivity disappears when SP is subjected to trypsin digestion, indicating its proteic nature (37). Further studies were performed to purify and characterize OIF in Bactrian camel (12, 38, 39), llamas (16, 40), and recently in dromedary camel (30). In the exhaustive study conducted by Pan et al. (38), proteins of Bactrian camel SP were compared with those of cattle, sheep, and swine using SDS-PAGE analysis. The smallest difference regarding the protein molecular weights was observed for a pair of proteins of camel and cattle SPs, with, respectively, 19.431 and 19.761 kDa. The 19.431 kDa proteic camel extract was eluted along a DEAE-cellulose column into seven fractions. Of all fractions injected in the muscle of female camels only the L_2_ fraction induced ovulation. Further L_2-2_ then L_2-2-2_ subfractions were identified according to their ability to induce ovulation following its intravenous injection in female camel and mice. This later L_2-2-2_ fraction of 8.360 kDa consisted of two components, the first named OIF_1_, represented a bioactive sequence of 19 amino acids, the second OIF_2_, represented a conserved sequence of 57 amino acids. Using similar approaches, Zhao et al. (12) and Li and Zhao (39) isolated proteic fractions (L3 and F3, F5, respectively) able to stimulate LH but not FSH release (Figure 2).

**Figure 3:**
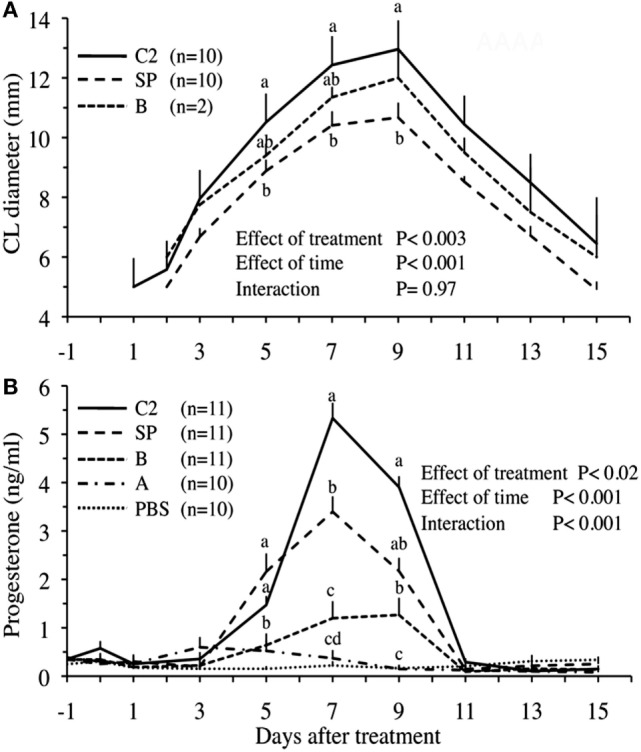
Corpus luteum (CL) diameter **(A)** and plasma progesterone concentrations **(B)** in llama females, which received different protein fractions separated from llama seminal plasma (SP). Administration concerned, whole SP (positive control), fractions A or B (isolated by hydroxylapatite column chromatography), fraction C2 (isolated by gel filtration chromatography), or phosphate-buffered saline (PBS, negative control). ^a,b,c,d^Within days, values with no common superscript are different (*P* < 0.05) [modified from Ratto et al. (40)].

In another series of experiments, Ratto et al. (16) attempted to inhibit OIF biological activity using the following: (1) filtration with nominal molecular mass cutoff −30°kDa, (2) enzymatic digestion with proteinase K, (3) filtration with charcoal dextran and heat treatment (12 h at 38°C or 1 h at 65°C), and (4) treatment with pronase E, a more aggressive enzyme. The treatment effect was challenged by testing intramuscular injections of resulting fractions in female llama. Llama ovulation bioassay indicated that OIF is a protein with a molecular mass larger than 30 kDa and that its bioactivity is inhibited by pronase E because such digestion resulted in proteins <15 kDa, but not by proteinase K even its digestion gave proteins <19 kDa (Table 1). In a follow-up study, Ratto et al. (40) isolated a protein C2 with a molecular mass of 13.221 kDa, which was still bioactive. This purified C_2_ was able to elicit a preovulatory LH surge followed by high rate of ovulation (>90%), similarly to SP. Furthermore, the CL attained a greater diameter and secreted more progesterone in C_2-_ than in SP-treated animals (Figure 3). Altogether, these results suggest that OIF is a large proteic complex made of different subunits with only a part being bioactive.

**Table 1:** Bioactivity of llama seminal plasma (SP) under different treatments in female llamas [modified from Ref. (16)].

	(A)						(B)		(C)			
	Effect of different molecular mass fractions of llama SP						Effect of SP treated with pronase E		Effect of SP treated with charcoal, proteinase K, or heat			
	≥30 kDa (*n* = 9)	10–30 kDa (*n* = 9)	5–10 kDa (*n* = 9)	<5 kDa (*n* = 9)	Whole seminal plasma (WSP) (*n* = 9)	Phosphate-buffered saline (PBS) (*n* = 9)	Untreated (*n* = 10)	Pronase E (*n* = 10)	Untreated(*n* = 7)	Charcoal (*n* = 7)	Proteinase K (*n* = 7)	Heat 65°C (*n* = 7)
Ovulation	9/9^a^	0/9^b^	0/9^b^	0/9^b^	9/9^a^	0/9^b^	9/10^a^	0/10^b^	7/7^a^	7/7^a^	7/7^a^	7/7^a^
Follicle diameter at treatment (mm)	–	–	–	–	–	–	9.7 ± 0.4^a^	9.0 ± 0.6^a^	10.4^a^ ± 0.9	10.8^a^ ± 0.9	9.3^a^ ± 0.2	9.0^a^ ± 0.7
Corpus luteum diameter (mm) on day 8 after treatment	–	–	–	–	–	–	–	–	10.5^a^ ± 0.5	11.3^a^ ± 0.7	11.7^a^ ± 0.9	11.5^a^ ± 0.8

*WSP (positive control), PBS (negative control). Day 0 = treatment*.

*^a,b^Proportions with different superscripts are different (*P* < 0.001)*.

*^a,b^Within rows, values with different superscripts are different (*P* < 0.01)*.

*^a^Within lines, no significant differences among groups*.

## Biochemical Nature of the OIF: β-NGF

Ratto et al. (17) further used different techniques to identify the biochemical nature of OIF from llama’s SP. They observed that the bioactive C_2_ fraction has an exact molecular mass of 13.221 kDa with 12–23 amino acid sequences similar to the human, porcine, bovine, and murine sequences of β subunit of nerve growth factor (β-NGF). Moreover, structure analysis and superposition of OIF composition with β-NGF of other species using X-ray diffraction revealed important structural similarities. They further found that the purified C_2_ fraction was able to induce neurite outgrowth and upregulated the mRNA expression of the NGF receptor tyrosine kinase receptor (TrKA) similarly to NGF treatment (17). Using another *in vivo* bioassay, they reported that injection of fraction C_2_ or mouse β-NGF leads to similar ovulation rate. Finally, they confirmed the presence of β-NGF in the SP of llamas using a western blot analysis with a polyclonal antibody against β-NGF. Altogether, this decisive study of Ratto et al. (17) pointed OIF as being β-NGF. Additional studies confirmed this observation. Thus, in alpaca an intramuscular administration of β-NGF induced ovulation in 80% of female (41), and β-NGF was found extremely abundant in alpaca and dromedary SP (41, 42). Furthermore, in this later species, recent purification of SP showed two protein peaks, P1 and P2. The P2 peak that contains a major protein with a molecular weight of 14 kDa was identified as Cam-β-NGF. Its intramuscular injection induced ovulation in 100% of females (30).

In addition to camelids, high concentrations of β-NGF have also been found in the SP of other induced ovulators. In rabbits, for example, β-NGF protein was found in the SP by western blot, and β-NGF mRNA was found in reproductive organs, including prostate, testis, and seminal glands (43–45). However, the ovulation-inducing effect of β-NGF was found weak in this species since injection of SP or murine β-NGF did not increase plasma LH-induced poor ovulation rates (43, 46, 47). Therefore, the significance of β-NGF in inducing ovulation may differ among induced ovulators.

β-NGF consists of a family of neurotrophin proteins, which play a role on neuron survival and differentiation in the peripheral and central nervous system and in the functional integrity of cholinergic neurons (48, 49). β-NGF is also largely present in peripheral tissues especially in salivary gland. The presence of β-NGF in male genital secretion is not a surprising finding since already in 1979, it was found in the guinea pig prostate (50) then in the prostate of rabbit, pig, and bull (51). Later, it was shown that bovine SP contains a high level of β-NGF (52) and that it can be produced by seminal vesicles (53) or epididymis (54).

The concentration of β-NGF is particularly high in the SP of llama where it reaches approximately 4–12 mg/ml, thus 15–30% of total protein (31, 41) whereas its concentration is 0.7 mg/ml in the bovine SP (52). Other studies confirmed that SP β-NGF concentration is greater in induced as compared to spontaneous ovulators. Such difference may explain the differential effect of β-NGF between these two categories of breeders. Indeed, llama’s SP induces ovulation in 78% of prepubertal mice (55) while purified β-NGF from cattle did not induce ovulation, although it accelerated the generation of a new follicular wave, influenced the dynamics in mature follicules and affected FSH and progesterone levels (56). Similarly, intramuscular injection of 2 ml of llama SP induced ovulation in 100% of llama females while 2 ml bull SP induced ovulation in only 26% of female llamas (15). Therefore, β-NGF is present in the SP of different mammalian species but at higher levels in induced ovulators.

## Mechanisms of Action of SP β-NGF: Local Versus Systemic Analysis

**Figure 4:**
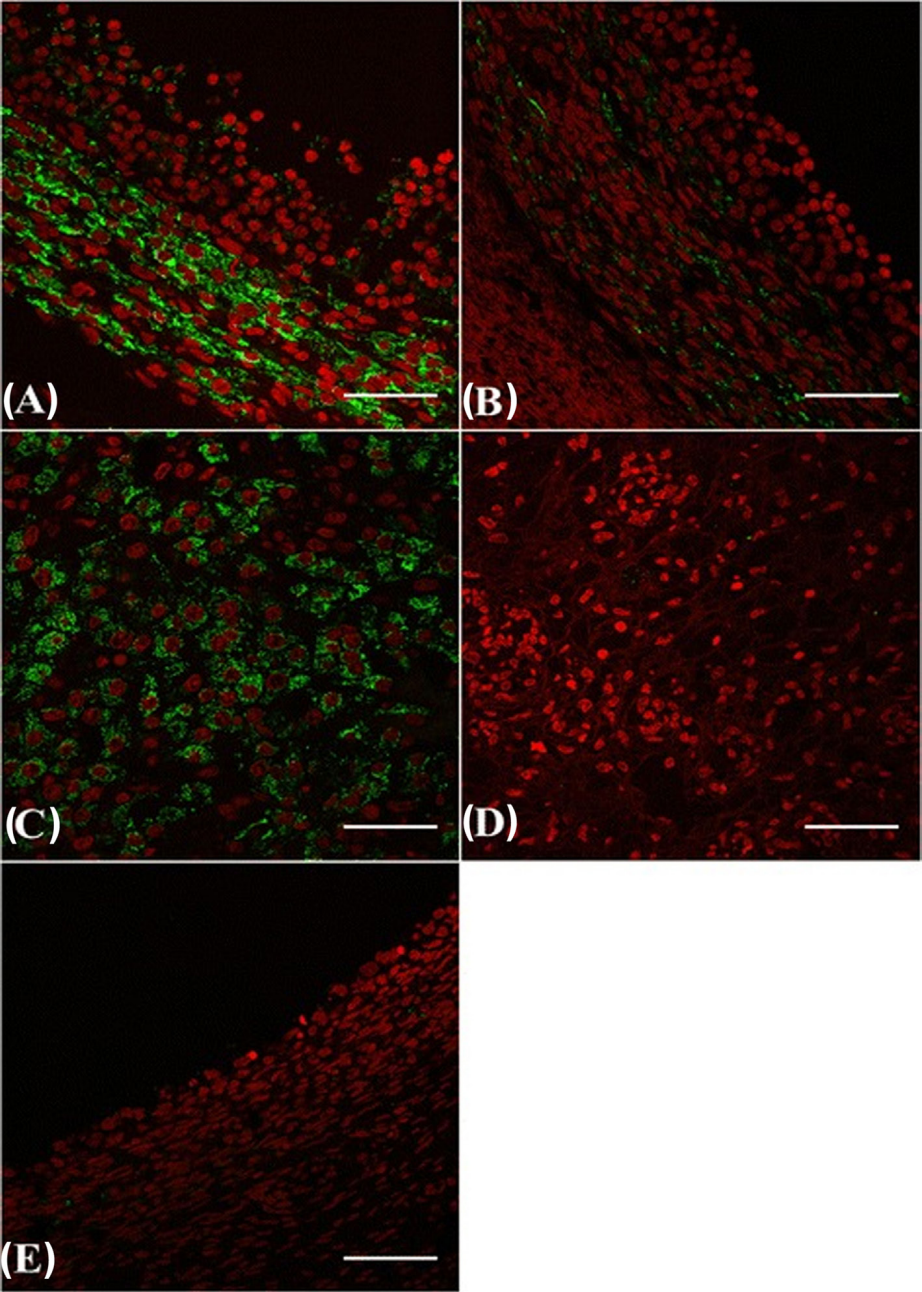
β-NGF-specific receptor TrkA immunofluorescence labeling (*green*) in the ovary [follicles and the corpus luteum (CL)] of cattle. Dominant follicle **(A)**, subordinate follicle **(B)**, CL **(C)**, CL from the previous cycle **(D)**, and regressing dominant follicle from the previous cycle **(E)**. *Red*: pseudo-color for nuclear counterstain. Scale bar = 50 µm [from Ref. (62)].

A local effect of β-NGF on ovaries was considered in spontaneous ovulators because it appears required for the development of primary and secondary follicles and for incorporating oocytes into the follicular structure, a process occurring independently of pituitary gonadotropins (48). In the female rat, β-NGF and its specific receptor (TrkA) are involved in the intracellular signaling pathways leading to follicular rupture (57–59) and both are upregulated in the theca cells of antral follicles during the preovulatory period, resulting in amplification of ovarian steroidogenesis (59). In addition, β-NGF, TrkA, and a non-specific receptors (p75) are found in fetal or adult ovaries of human (60), mice (48), and guinea pig (61). Furthermore, recent data in cattle suggest that β-NGF exerts its luteotrophic effect by acting locally at the level of ovaries through TrkA located in the dominant follicle and the developing of CL [Figure 4; (36, 62)]. These observations are in favor of a significant role of β-NGF on ovaries of several species.

**Figure 5:**
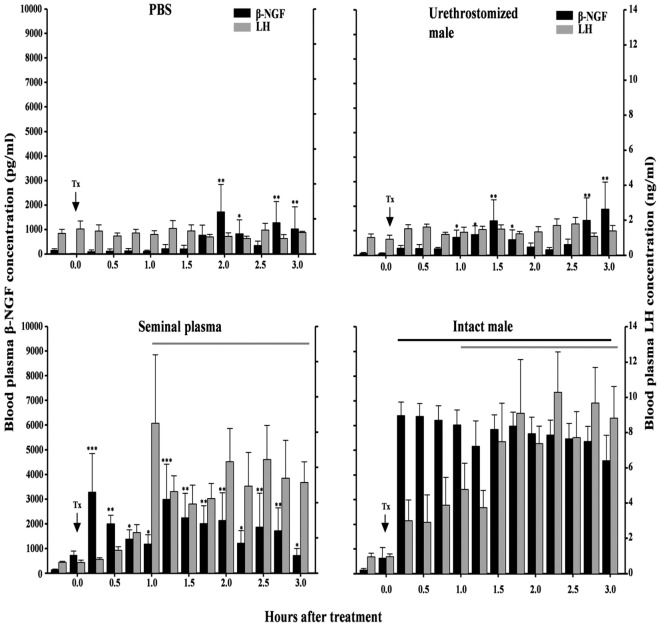
Effect of intrauterine infusion of either phosphate-buffered saline (PBS) (*n* = 5) or seminal plasma (*n* = 6), or after mating with an urethrostomized (*n* = 5) or intact male (*n* = 6) on plasma β-NGF and luteinizing hormone (LH) concentrations in llamas. Interval during which plasma LH and β-NGF concentration differed from pretreatment values versus pretreatment levels. Tx, treatment [from Ref. (21)].

In female alpaca, the ovulation rate depends on the dose and site of deposition of SP (14, 36). Indeed, ovulation rate is 0% after 1 ml infusion in the uterine body (41% after 2 ml infusion in the uterine horns; 67% if an endometrial curettage is made before intrauterine deposition and 93% after an intramuscular injection) (Table 2). These findings suggest that genital mucosa may prevent β-NGF absorption. In this regard, copulation in alpacas and llamas is a 30–50 min long event with an intrauterine ejaculation suggesting that in natural mating β-NGF absorption from SP is probably facilitated by the hyperemia of the excoriated endometrium resulting from repeated abrasion by penis (14, 19, 28, 63). Taken together, these findings are consistent with the hypothesis that β-NGF exerts its effect *via* a systemic rather than a local route, an hypothesis confirmed by the study of Berland et al. (21). In this study, blood plasma β-NGF level was measured in four groups of female llamas as follows: (1) receiving intrauterine infusion of SP, (2) receiving intrauterine infusion of PBS, (3) mated to an intact male, and (4) mated to urethrostomized male. Female llamas mated with an intact male or receiving an intrauterine infusion of SP showed a large increase in circulating β-NGF already 15 min after treatment while the two other groups displayed low β-NGF levels (Figure 5). Authors also reported that ovulation depended on this elevated plasmatic β-NGF levels as only female mated with an intact male or given intrauterine infusion displayed a preovulatory surge of LH and ovulated. These new findings are very important since they demonstrated that SP β-NGF deposited in the uterus is absorbed within the general circulation and is possibly conveyed toward the central nervous system to induce a preovulatory LH surge.

**Table 2:** Endometrial curettage effect on the ovulation rate and the formation of follicle and corpus luteum (CL) in female alpacas after intrauterine infusion with alpaca seminal plasma (SP).

	Intramuscular		Intrauterine		Intrauterine with curettage	
	SP	Phosphate-buffered saline (PBS)	SP	PBS	SP	PBS
Follicle diameter at treatment (mm)*	8.0 ± 0.3 (*n* = 15)	8.2 ± 0.3 (*n* = 15)	8.1 ± 0.3 (*n* = 17)	8.0 ± 0.3 (*n* = 15)	8.3 ± 0.2 (*n* = 15)	8.4 ± 0.3 (*n* = 15)
Ovulation rate (%)	14/15^a^ (93%)	0/15^c^ (0%)	7/17^b^ (41%)	0/15^c^ (0%)	10/15^a,b^ (67%)	0/15^c^ (0%)
CL diameter (mm) on day 8 (day 0 = Treatment)*	9.3 ± 0.4 (*n* = 4)	–	9.5 ± 0.3 (*n* = 7)	–	9.4 ± 0.4 (*n* = 10)	–

*Data are presented as mean ± SEM (14)*.

*No difference among groups (*P* ≥ 0.9).

*^a,b,c^Proportions with different superscript are different (*P* < 0.001)*.

## Identification of the β-NGF Central Target(s): The Kp Neuron Hypothesis

Circulating β-NGF can induce LH secretion by a direct effect on the pituitary gonadotrophs or upstream in the central structures like GnRH neurons. Direct effect of β-NGF on cultured pituitary cells has been reported in different species. Indeed, incubation of gonadotrophic cells with whole semen, SP or purified β-NGF induced a significant increase in LH secretion (39, 64, 65). The obtained effect seems to be not mediated by GnRH receptors since the LH secretion from rat pituitary cells was not impaired by an anti-GnRH antibody in the media (64). These *in vitro* results indicate that β-NGF plays its role independently from GnRH as also supported by the presence of β-NGF TrkA receptors in gonadotrophs cells (66). Furthermore, *in vivo* studies conducted in llamas and alpaca showed that LH secretion was more sustained in females treated with SP or purified β-NGF than in the ones treated with GnRH (13, 31, 34, 40). Furthermore, β-NGF pretreatment of llamas with a GnRH antagonist (Cetrorelix) suppressed the β-NGF- and GnRH-induced LH secretion and subsequent ovulation, thus demonstrating that circulating β-NGF is acting upstream of the pituitary GnRH receptors (67).

In rat, β-NGF receptors are found in the hypothalamus, particularly in the medial preoptic area (POA), arcuate nucleus, and ventral premammillary nucleus (68, 69). In llama also, Adams et al. (36) reported that β-NGF and TrkA are present in the hypothalamus. Other studies have provided indirect evidences for the involvement of hypothalamus in the β-NGF-induced LH release. The study conducted by Silva et al. (70) investigated the role of estradiol (for which a high concentration in the hypothalamus is required to induce the LH surge) in mediating β-NGF effects. Indeed, authors showed that the β-NGF-induced LH surge was suppressed in ovariectomized female llamas but partly restored by estradiol administration, therefore indicating that β-NGF effect requires the hypothalamic action of estradiol.

It is now well established that the main hypothalamic sites of action of estradiol are Kp neurons. Since the discovery in 2003 that human (71) and mice (72) lacking functional Kp receptor (Kiss1r) are infertile, numerous studies have demonstrated the critical role of this neuropeptide in pubertal development and adult reproduction [see Ref. (1) for review]. Kp neurons have been found in the hypothalamus of all mammals investigated so far, and they project their fibers mainly to the GnRH cell bodies, in the POA, and nerve terminals, in the median eminence (73–77). All Kp neurons express estradiol receptors, and they are now considered as the main central sites for both the positive and negative (according the hypothalamic nuclei where Kp neurons are located) feedback effects of estradiol. Kp is extremely potent to trigger GnRH release and the downstream LH and FSH secretion, and it is now accepted that Kp is responsible for the induction of the preovulatory LH surge in female mammals (78).

**Figure 6:**
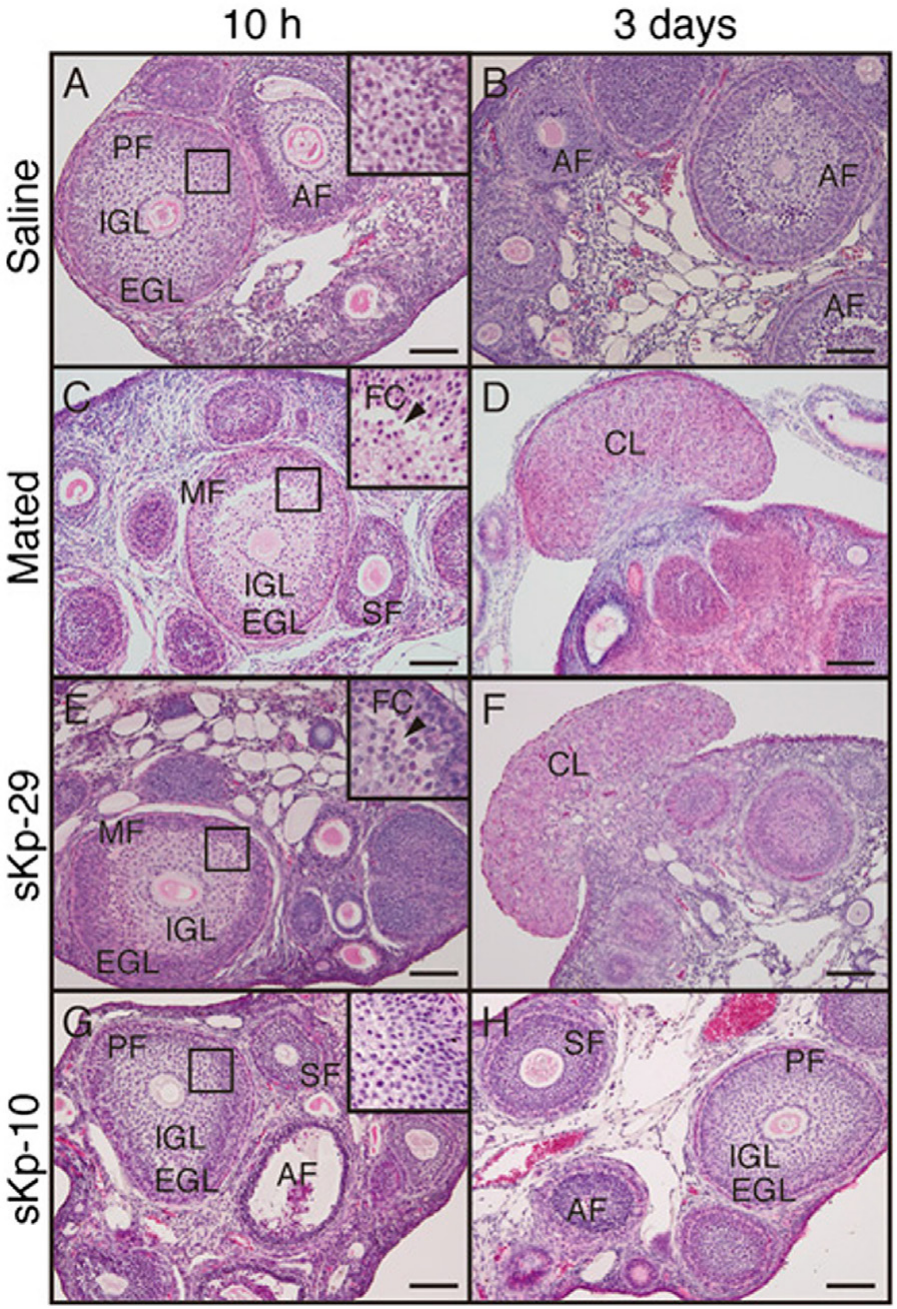
Effects of kisspeptin (sKp-29 and sKp-10) injections on development and formation of follicular and CL in an induced ovulator, the *musk shrew*. Photomicrographs are of classical histology of ovaries stained with H&E. **(C,D)** Females mated, **(A,B)** injected with saline, **(E,F)** injected with sKp-29, **(G,H)** sKp-10. Photographs were taken 10 h **(A,C,E,G)** or 3 days **(B,D,F,H)** after the onset of mating or injection. A slit-like FC (arrowheads) in ovarian follicles at 10 h and fungiform corpora lutea in the ovary at 3 days were observed in mated and sKp-29-injected females. Insets show the boxed area in each panel at higher magnification. AF, atretic follicle; CL, corpus luteum; EGL, extra granulosa layer; FC, follicular cavity; IGL, inner granulosa layer; MF, mature follicle; PF, premature follicle; SF, secondary follicle. Scale bars: 100 µm [from Ref. (79)].

**Figure 7:**
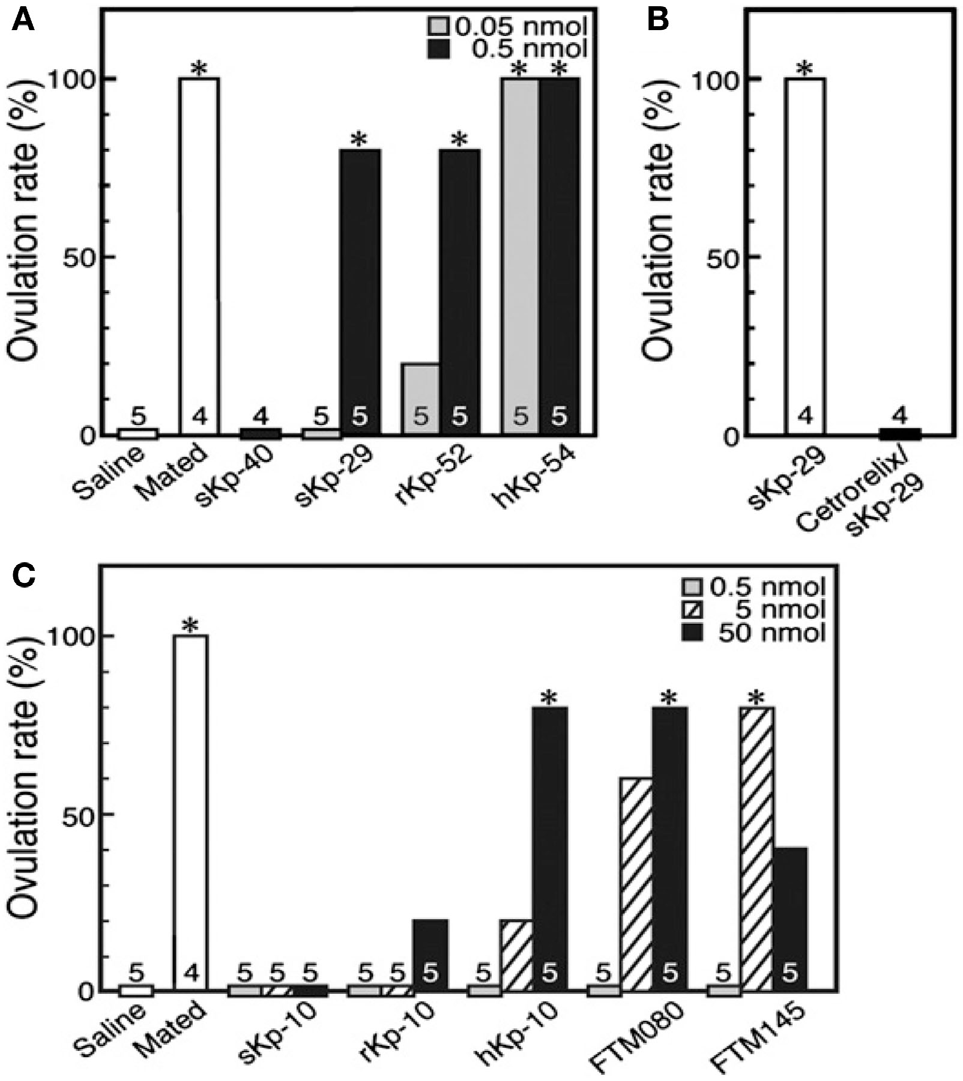
The ovulation rate in musk shrews, an induced ovulator, under the effect of various kisspeptins and GPR54 agonists. **(A)** Females mated or injected with saline or sKp-40 (0.5 nmol per animal) or with sKp-29, rKp-52, or hKp-54 (0.05 or 0.5 nmol per animal). *P < 0.05. **(B)** Effect of cetrorelix, a gonadotropin-releasing hormone antagonist, on sKp-29-induced ovulation. *P < 0.05. **(C)** Females mated or injected with saline, sKp-10, rKp-10, hKp-10, or the GPR54 agonists FTM080 or FTM145 (0.5, 5, or 50 nmol per animal). *P < 0.05. Number of animals treated are shown over each column [from Ref. (79)].

Recent studies have investigated the putative role of Kp in induced ovulators. In the musk shrew, the number of Kp expressing neurons is regulated by estradiol, and injection of exogenous suncus Kp (sKp-29) was found to induce follicular maturation and ovulation similarly than matting [Figure 6; (79)]. Furthermore, Kiss1r mRNA was found in the musk shrew hypothalamus and pretreatment with a GnRH antagonist completely blocked the sKp-29-induced ovulation (Figure 7) suggesting that Kp induces ovulation *via* an activation of GnRH neurons.

**Figure 8:**
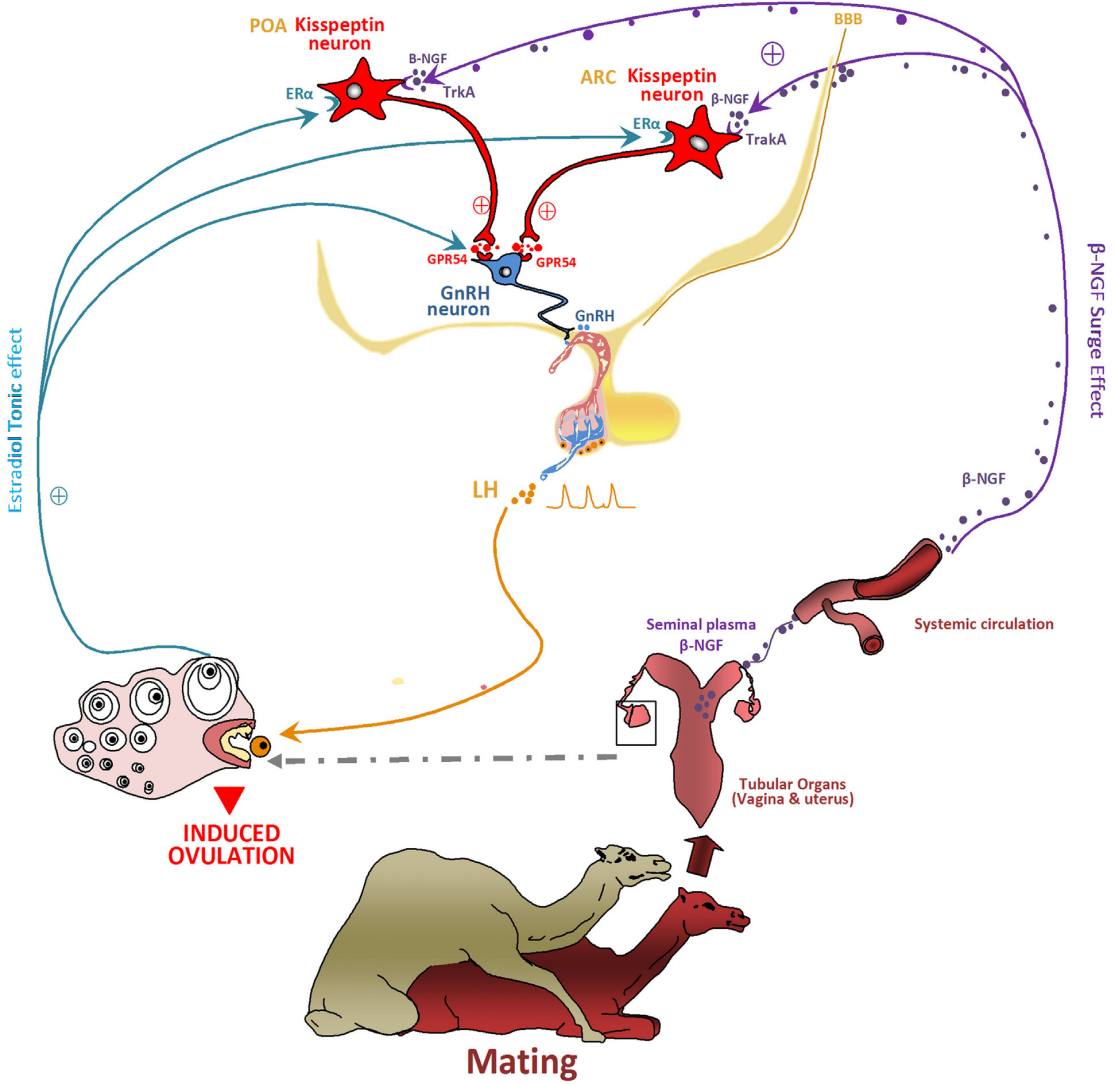
Proposed model hypothesis for kisspeptin (Kp) neurons involvement in the β-NGF initiation of the preovulatory luteinizing hormone (LH) surge and induced ovulation following mating in camelids. The figure shows how Kp neurons may act as central processors for relaying β-NGF/matting signals to gonadotropin-releasing hormone (GnRH) neurons activation. Following coitus, the β-NGF of the seminal plasma is absorbed through the genital mucosa within the general circulation and conveyed toward the central nervous system. β-NGF crosses probably the blood–brain barrier (BBB) to reach the hypothalamus at the level of preoptic area (POA) and arcuate nucleus (ARC) where it binds the TrkA receptor expressed by Kp neurons. By activating Kp neurons, β-NGF induces a sequence of neuroendocrine events leading to the ovulation: Kp released at axonal ends binds the GPR54 receptors located on the GnRH neurons and stimulate the release of GnRH. The GnRH on the other hand stimulates the release of a pulse of LH, which leads to the rupture of the dominant follicle and thus induced the ovulation. In such seasonal breeders, Kp would be also responsible, as for other species, for the seasonal stimulating of the neuroendocrine reproductive axis. The known steroids positive feedback responsible of ovulation in spontaneous ovulators is lacking in reflex ovulators, However, estradiol seems to play an important role for inducing ovulation in such species as discussed before in the musk shrew and llama. It seems to be involved in β-NGF effect. Estradiol would be responsible of a tonic effect while the β-NGF exerts a surge effect. By binding its receptor ERα located on Kp neurons, estradiol promotes the neuronal activation and the release of Kp. The effect of Kp would be also distinct and unlike for the two Kp populations of POA and ARC.

From these data, it is tempting to speculate that Kp neurons may be one of the central β-NGF targets for the mating-induced ovulation. Indeed, in the musk shrew, mating induces a large increase in c-Fos expression (marker of neuronal activation) in hypothalamic Kp neurons (79). This finding indicates that β-NGF may act through the hypothalamic Kp neurons to elicit the preovulatory LH surge and may be similarly important in the spontaneous and induced ovulators to induce the LH surge and ovulation. This hypothesis raises the question of the route by which the mating-induced increase in circulating β-NGF reaches the female hypothalamus. Adams et al. (36) suggested that the cerebrospinal fluid may be a potential route for the central effect of β-NGF, an hypothesis requiring that circulating β-NGF crosses the blood–brain barrier (BBB). Indeed, Loy et al. (80) observed a specific uptake of intravenously injected I^125^-NGF into different parts of central nervous system 1 h after injection, an observation further confirmed in mice (81) and rabbits (82). Thus, these data indicate that β-NGF, released in the general circulation following induced ovulator mating, can cross the BBB, and it therefore may reach hypothalamic cells, possibly Kp or GnRH neurons to further trigger the preovulatory LH surge (Figure 8).

Taken together, these results have demonstrated the pivotal role played by β-NGF in the modulation and activation of the reproductive axis of induced ovulators. However, its site and mechanism of actions are far from being understood. In this regard, the Kp hypothalamic neurons appear as an interesting putative target, an hypothesis that now required further investigation in induced ovulators, especially camelids.

## Author Contributions

KA and VS conceived and designed the hypothesis. KA, NB, HA, and VS wrote and approved the final review.

## Conflict of Interest Statement

The authors declare that the research was conducted in the absence of any commercial or financial relationships that could be construed as a potential conflict of interest.
